# Association between high-resolution MRI-detected extramural vascular invasion and tumour microcirculation estimated by dynamic contrast-enhanced MRI in rectal cancer: preliminary results

**DOI:** 10.1186/s12885-019-5732-z

**Published:** 2019-05-27

**Authors:** Yan Chen, Xinyue Yang, Ziqiang Wen, Yiyan Liu, Baolan Lu, Shenping Yu, Xiaojuan Xiao

**Affiliations:** 1 Department of Radiology, the First Affiliated Hospital, Sun Yat-sen University, Guangzhou, 510080 China; 2 Department of Radiology, Zhujiang Hospital of Southern Medical University, Guangzhou, 510282 China; 3 Department of Radiology, the Eighth Affiliated Hospital, Sun Yat-sen University, Shenzhen, 518036 China

**Keywords:** Rectal cancer, Extramural vascular invasion, Dynamic contrast-enhanced MRI, Tumour microcirculation

## Abstract

**Background:**

To determine whether magnetic resonance imaging (MRI)-detected extramural vascular invasion (mrEMVI) status is associated with quantitative perfusion parameters derived from dynamic contrast-enhanced MRI (DCE-MRI) in rectal cancer.

**Methods:**

Seventy-two patients with rectal adenocarcinoma who underwent curative surgery alone within 2 weeks following rectal MRI were enrolled in this retrospective study. mrEMVI status was determined based on high-resolution MRI. The quantitative perfusion parameters (*K*^*trans*^, *k*_*ep*_ and *v*_*e*_) derived from DCE-MRI were calculated from all sections containing tumours. DCE-MRI parameters and clinicopathological variables in patients with different mrEMVI statuses were compared.

**Results:**

For patients who were mrEMVI positive, the tumours demonstrated significantly lower *k*_*ep*_ values (*P* = 0.012) and higher *v*_*e*_ values (*P* = 0.021) than tumours of patients who were mrEMVI negative, while the *K*^*trans*^ value displayed no significant difference (*P* = 0.390). The patients who were mrEMVI positive had larger tumour size, higher pathological tumour stage and increased regional nodal metastases compared to those who were mrEMVI negative (2.9 cm vs. 3.5 cm, *P* = 0.011; 63.8% vs. 92.0%, *P* = 0.010; 36.2% vs. 76.0%, *P* = 0.001; respectively).

**Conclusions:**

This study demonstrated for the first time that tumour microcirculation is altered in mrEMVI-positive patients with rectal adenocarcinoma, as evidenced by significantly lower *k*_*ep*_ and higher *v*_*e*_ values. In addition, these patients were more likely to have a larger tumour size, a higher pathological tumour stage and regional nodal metastases than mrEMVI-negative patients.

## Background

Extramural vascular invasion (EMVI), defined histologically as the presence of tumour cells within blood vessels outside the muscularis propria of the rectal wall, is associated with a higher risk of local and distant recurrence and poorer survival [1, 2]. Although dissemination of tumour cells into small vessels (< 3 mm in diameter) can be histologically confirmed, this identification is of little clinical importance [2, 3]. In addition, when involved vessels are extensively destroyed beyond recognition, they are underreported by pathologists [4]. Furthermore, the reported detection rates of EMVI vary widely due to heterogeneity in staining, the extent of histopathological evaluation or diagnostic criteria for EMVI [1, 4, 5]. High-resolution magnetic resonance imaging (MRI), generally consisting of thin sections (3 mm) with an in-plane resolution of 0.5–0.8 mm, is the primary imaging modality for preoperative local staging of rectal cancer [6]. This method has shown accuracy in identifying EMVI, especially in vessels greater than 3 mm in diameter [3, 7, 8]. In addition, several studies have suggested that MRI-detected EMVI (mrEMVI) is a strongly predictive of poor prognosis [9, 10]. Therefore, the identification of EMVI based on high-resolution MRI can be regarded as more clinically relevant than histological identification.

Tumour angiogenesis, which is regulated by numerous factors, is a fundamental process in the growth and invasion of rectal cancer [11]. Among the most critical regulator of angiogenesis is vascular endothelial growth factor (VEGF) [12]. Microvessel density (MVD), which is a measurement of the density of microvessels, has been found to be correlated with VEGF expression, and both of these immunohistochemical markers are widely used to evaluate tumour angiogenesis [13, 14]. Immunohistochemistry is invasive, time consuming and has poor measurement repeatability, whereas dynamic contrast-enhanced MRI (DCE-MRI) is a promising method to noninvasively evaluate tumour microcirculation in vivo. Kinetic parameters estimated from DCE-MRI are able to assess tumour blood flow, capillary permeability and permeability surface area, which may reflect tumour biology. The three principle parameters are the volume transfer constant between blood plasma and extravascular extracellular space (EES) (*K*^*trans*^, min^− 1^), the rate constant between EES and blood plasma (*k*_*ep*_, min^− 1^) and the volume of EES per unit volume of tissue (*v*_*e*_, 0 < *v*_*e*_ < 1) [15].

Neovascularization usually has an immature vascular structure that can be easily infiltrated by tumour cells [16, 17]. However, the relationship between EMVI status and tumour microcirculation in patients with rectal cancer has not been studied previously. Hence, this study aims to investigate whether patients with different mrEMVI statuses show any difference in DCE-MRI parameters (*K*^*trans*^, *k*_*ep*_ and *v*_*e*_), with the ultimate aim of identifying a potential relationship between mrEMVI status and tumour microcirculation.

## Methods

### Patients

This retrospective study was conducted from November 2014 to December 2015 after approval by our institutional review board. Written informed consent was obtained from all participants included in the study.

**Fig. 1:**
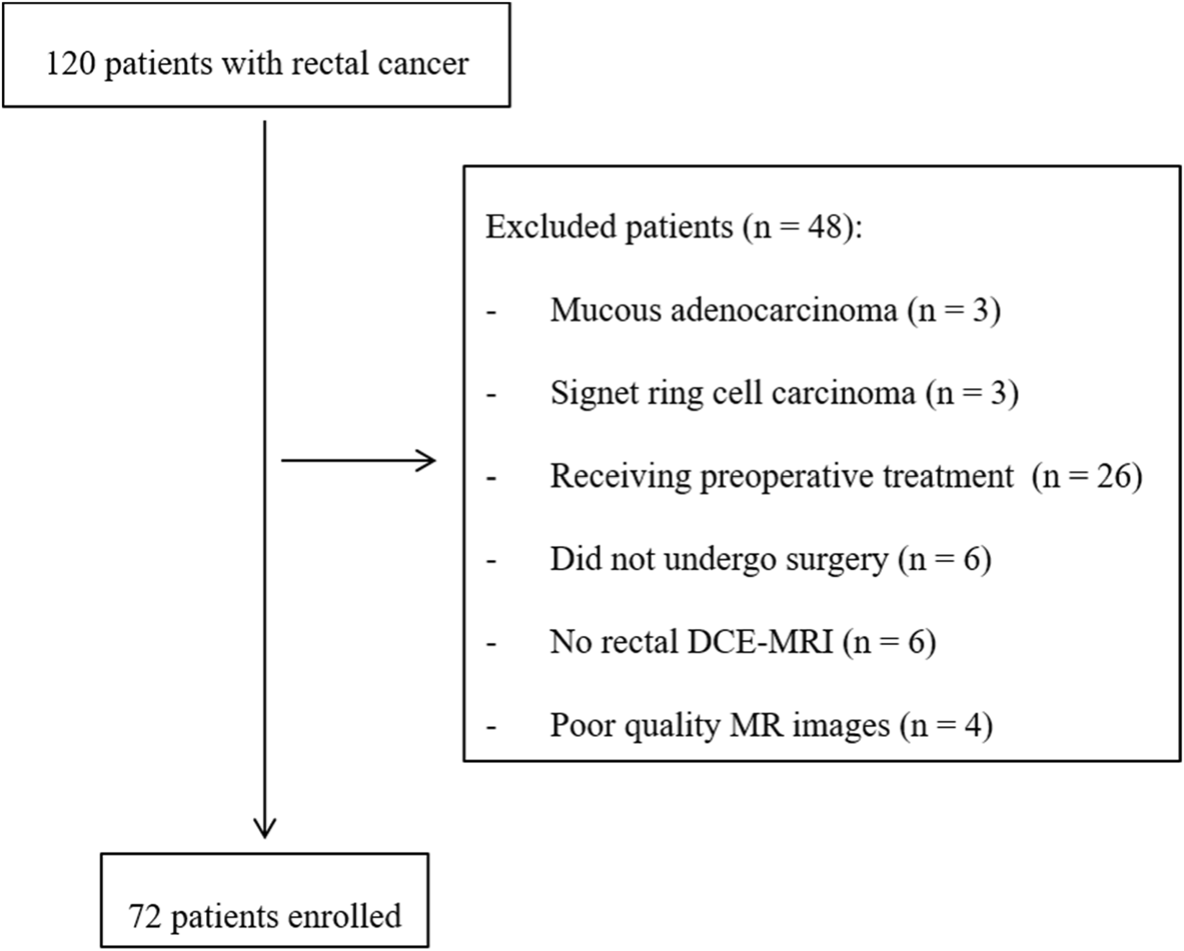
Flow diagram of the study patients

Patients with histologically proven primary rectal adenocarcinoma who underwent curative surgery (R0) for rectal cancer (including anterior resection and abdominoperineal resection) within 2 weeks after rectal high-resolution MRI and DCE-MRI were enrolled. A total of 120 consecutive patients with proven rectal cancer had preoperative rectal MRI. Among them, 48 patients were excluded for the following reasons: proven special histopathological type, including mucous adenocarcinoma (*n* = 3) and signet ring cell carcinoma (n = 3); receiving preoperative radiotherapy, chemotherapy or chemoradiotherapy (*n* = 26); did not undergo surgery (*n* = 6); no rectal DCE-MRI (n = 6); and poor quality MR images (*n* = 4). Ultimately, 72 patients (41 males and 31 females; mean age, 60 ± 10 years) were included (Fig. 1).

### MRI examination

An appropriate amount (20–80 mL) of ultrasonic gel was administered in the rectum, except for patients with low or large rectal tumours. To reduce bowel peristalsis, 20 mg of raceanisodamine hydrochloride was injected intramuscularly approximately 10 min before the MRI. In addition, the patients were asked to urinate to prevent the effects of urine inside the bladder on DCE-MRI data postprocessing.

Imaging was performed using a 3.0-Tesla MR scanner (Magnetom Verio, Siemens Healthcare, Erlangen, Germany) with a 6-element body phased-array coil. The coil centre was placed at the level of the pubic symphysis and adjusted according to the tumour location. All of the patients were placed in the supine position with a feet-first orientation.

The MRI protocols included the following: (a) sagittal, coronal and oblique axial high-resolution T2-weighted imaging (T2WI) using a turbo spin-echo sequence with the oblique axial plane perpendicular to the tumour basement and (b) oblique axial DCE-MRI using a three-dimensional dynamic T1-weighted sequence based on a time-resolved technique with interleaved stochastic trajectories (TWIST). After five precontrast phases, DCE-MRI was simultaneously initiated at the time of an intravenous bolus injection of 0.1 mmol/kg gadopentetate dimeglumine at a rate of 3.0 mL/s with a power injector, followed by a 25 mL saline flush. A total of 75 consecutive dynamic images with a temporal resolution of 4.25 s were acquired. In addition, a volume-interpolated body examination (VIBE) sequence with a dual-flip-angle was acquired to perform T1 mapping in an oblique axial plane before contrast agent administration. The detailed parameters of the protocols are listed in Table 1.

**Table 1:** MRI protocols for rectal cancer

Protocols	TR/TE (ms)	Slice thickness (mm)	Distance factor (%)	Slices	Flip angle (°)	Base resolution	Phase resolution (%)	FOV (mm)	Voxel size (mm)	Time acquisition
Sagittal T2WI	3000/87	3.0	0	19	150	320	80	180	0.7 × 0.6 × 3.0	2 min 30 s
Coronal T2WI	4000/77	3.0	0	25	137	384	80	220	0.7 × 0.6 × 3.0	2 min 52 s
Oblique axial T2WI	3000/84	3.0	0	24	150	320	100	180	0.6 × 0.6 × 3.0	3 min 18 s
Oblique axial T1 mapping	5.08/1.74	3.6	20	20	2 and 14	192	72	260	1.9 × 1.4 × 3.6	2 min 29 s
Oblique axial DCE-MRI	4.83/1.87	3.6	20	20	12	192	69	260	2.0 × 1.4 × 3.6	5 min 24 s

*TR* repetition time, *TE* echo time, *FOV* field of view, *T2WI* T2-weighted imaging, *DCE-MRI* dynamic contrast-enhanced magnetic resonance imaging

### Imaging analysis

#### mrEMVI detection

Two radiologists specialized in rectal MRI with 6 and 8 years of experience independently reviewed high-resolution T2WI for the presence of mrEMVI. Both radiologists were blinded to the DCE-MRI results and histopathological findings. Imaging findings suggestive of mrEMVI were serpiginous extension of tumour signal into extramural vessels contiguous with or separated from the primary rectal tumour that resulted in expansion and/or irregularity of these vessels [3, 18]. Discrepant diagnoses were resolved by a third radiologist with 23 years of experience of rectal MRI for a consensus or majoritarian decision.

#### DCE-MRI data postprocessing

**Fig. 2:**
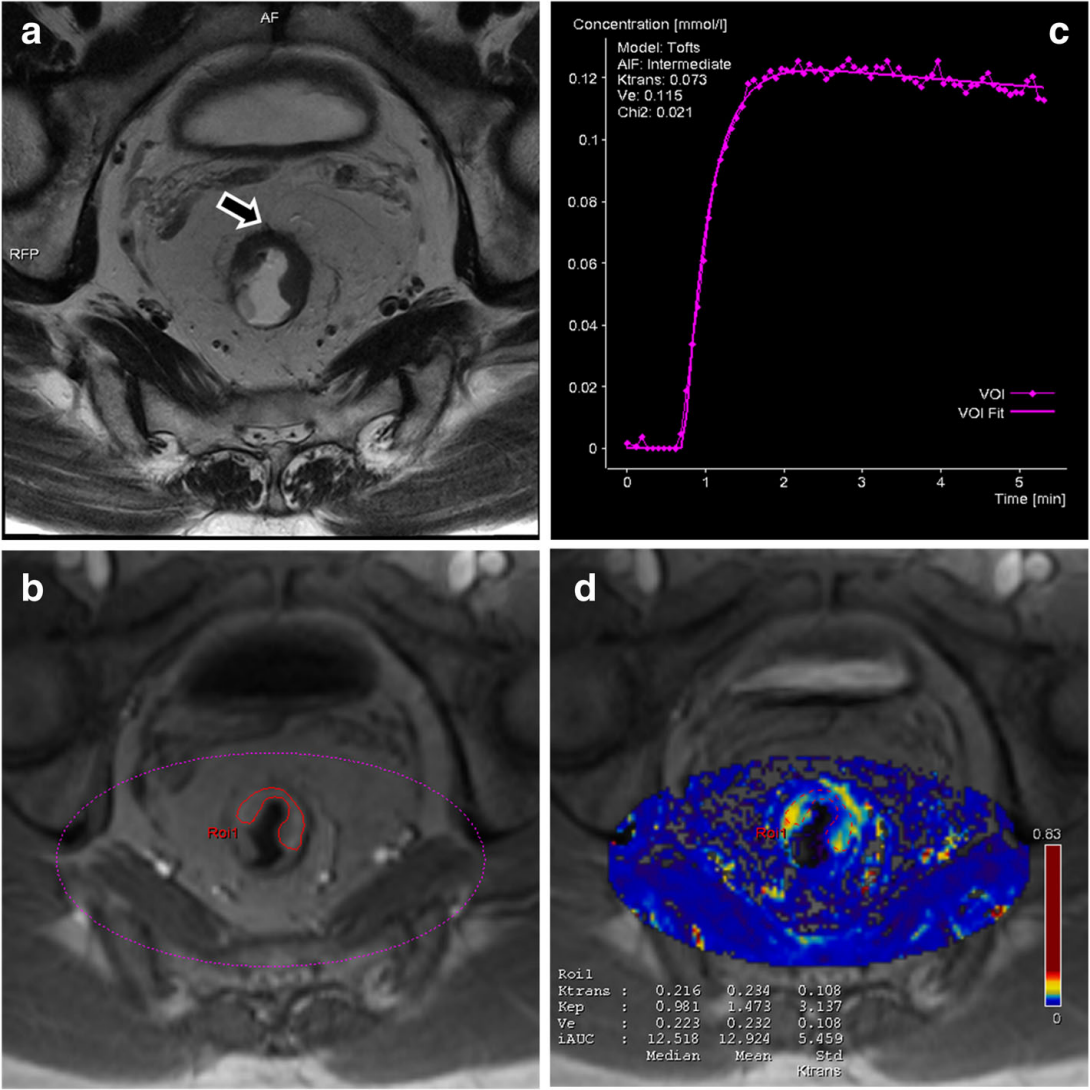
A patient with rectal adenocarcinoma (pT3N1, moderately differentiated). **a** Oblique axial high-resolution T2WI depicted the extramural vessels were in the vicinity of stranding rectal tumour, but these vessels had normal calibre, and there is no definite tumour signal within the vessels (arrow), indicating mrEMVI-negative status. **b** A VOI (pink dashed line) was selected on DCE-MRI, and (**c**) the AIF with the smallest chi-square value was than selected. **b** The ROI (red continuous line) was drawn along the edge of the tumour guided by corresponding (**a**) oblique axial high-resolution T2WI. **d** The ROI was copied to *K*^*trans*^ map, and the mean *K*^*trans*^ (0.234 min^− 1^), *k*_*ep*_ (1.473 min^− 1^) and *v*_*e*_ (0.232) values were obtained

**Fig. 3:**
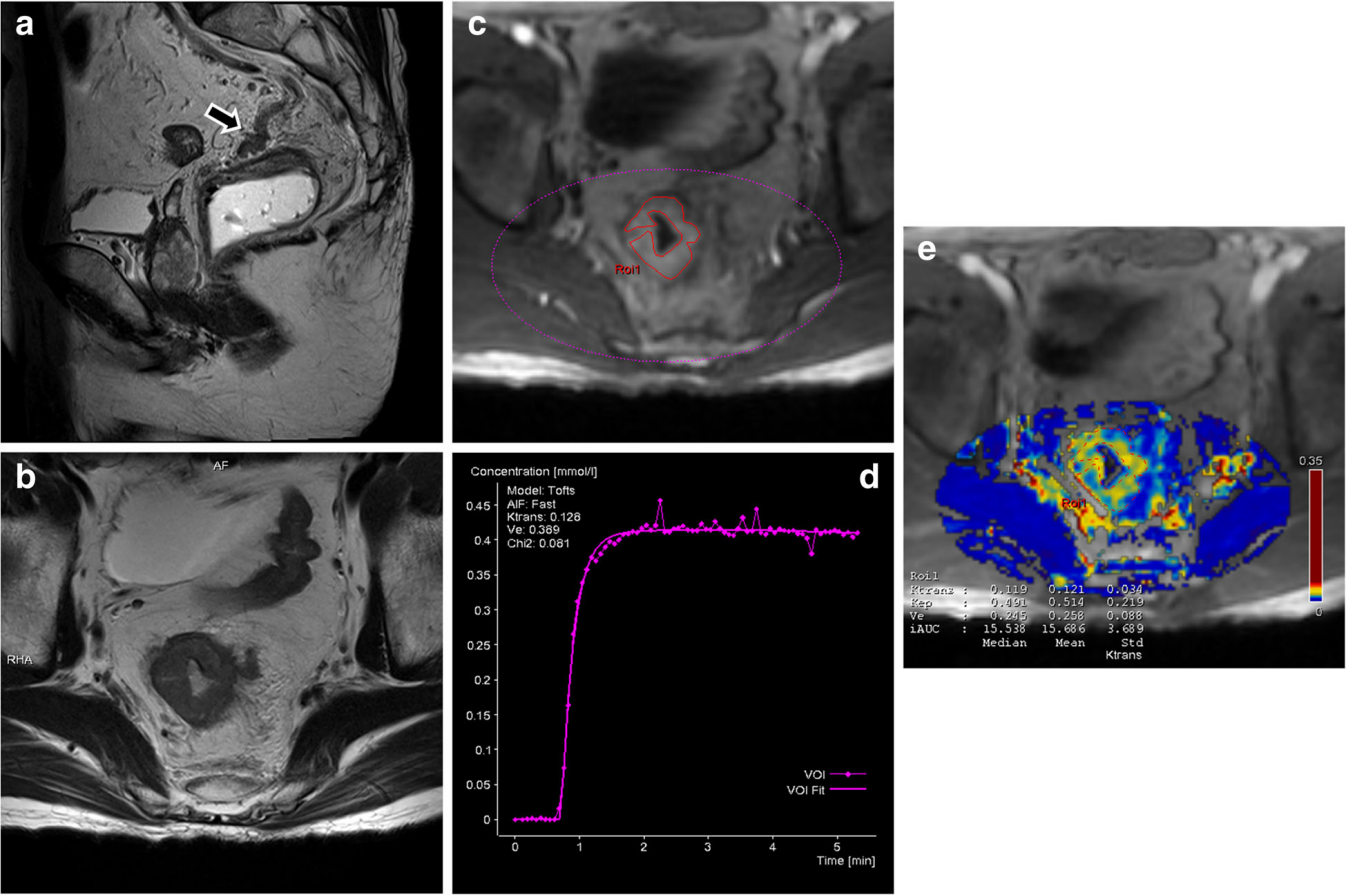
A patient with rectal adenocarcinoma (pT4N1, moderately differentiated). **a** Sagittal high-resolution T2WI depicted definitive tumour signal within a marked expanded and irregular extramural vessel (arrow), confirming mrEMVI-positive status. **c** A VOI (pink dashed line) was selected on DCE-MRI, and (**d**) the AIF with the smallest chi-square value was the selected. **c** An ROI (red continuous line) was drawn along the edge of the tumour guided by corresponding (**b**) oblique axial high-resolution T2WI. **e** The ROI was copied to *K*^*trans*^ map, and the mean *K*^*trans*^ (0.121 min^− 1^), *k*_*ep*_ (0.514 min^− 1^) and *v*_*e*_ (0.258) values were obtained

At one-month intervals, DCE-MRI data were independently postprocessed by the same two radiologists (with 6 and 8 years of experience of rectal MRI) on an MRI workstation (Syngo MultiModality Workplace, VE40A, Siemens Healthcare, Erlangen, Germany) using Tissue 4D software. First, the T1 mapping was registered to the motion-corrected DCE-MRI images. Second, a volume of interest (VOI) was manually drawn on DCE-MRI, and the VOI was kept away from bladder. To calculate the parametric map of *K*^*trans*^ in the VOI, an arterial input function (AIF) with the smallest chi-square value was selected. Third, regions of interest (ROIs) were manually contoured along the edges of the tumours on all slices containing tumour on DCE-MRI guided by corresponding oblique axial high-resolution T2WI. Lastly, the ROIs were copied to *K*^*trans*^ map, and the mean quantitative perfusion parameters (*K*^*trans*^, *k*_*ep*_ and *v*_*e*_) were generated from a Tofts two-compartment model [15] (Figs. 2 and 3). The parameters were averaged to calculate the mean values of the whole tumour volume, and the parameters were averaged between the two radiologists for further analyses.

### Histopathological and synchronous distant metastasis assessment

The haematoxylin and eosin stained slides from all resected specimens were assessed to confirm the tumour and node stage and tumour differentiation. When the tumour was limited within muscularis propria, the stage was categorized as T1/T2; when the tumour had invaded through the muscularis propria into the perirectal tissue, the stage was categorized as T3/T4. All of the lymph nodes were analysed, and malignancy was defined when tumour cells were observed within the node under a light microscope. The histopathological stage of rectal cancer was determined according to the 7th American Joint Committee on Cancer TNM manual [19].

Distant metastatic lesions detected at the initial diagnostic workup and within a 6-month follow-up after the initial diagnosis were considered synchronous distant metastases. When distant metastases were suspected on initial contrast-enhanced abdominal-pelvic and chest computed tomography (CT), contrast-enhanced liver MRI or fludeoxyglucose-positron emission tomography/computed tomography (FDG-PET/CT) were used for more precise evaluation. The distant metastatic lesions were confirmed by histopathology or by sequential 6-month radiologic follow-up.

### Statistical analysis

A Kolmogorov-Smirnov test was conducted to evaluate the data distribution. Normally distributed data were expressed as the means with standard deviations (SDs), while non-normally distributed data were reported as medians with ranges. Normally distributed data were analysed using independent samples *t*-test, while non-normally distributed data were analysed using Mann-Whitney *U* test. Categorical data were compared with *χ*^*2*^ or Fisher’s exact tests. A two-sided *P* value < 0.05 was considered statistically significant. The above statistical analyses were performed using SPSS software (version 20.0).

Interobserver agreement for mrEMVI status evaluation between the two radiologists was analysed using Kappa statistics (0.00–0.20 poor, 0.21–0.40 fair, 0.41–0.60 moderate, 0.61–0.80 good and 0.81–1.00 excellent agreement) with a 95% confidence interval (CI) [20]. Interobserver agreement for DCE-MRI quantitative perfusion parameters measurements between the two radiologists was analysed by calculating the intraclass correlation coefficient (ICC) (0.00–0.20 poor, 0.21–0.40 fair, 0.41–0.60 moderate, 0.61–0.80 good and 0.81–1.00 excellent correlation) with a 95% CI and according to the method of Bland and Altman [21]. The above statistical analyses were performed using MedCalc software (version 15.8).

## Results

### Clinicopathological characteristics

The median duration between MRI and curative resection was 5 days (range: 1–14 days). Table 2 summarizes the clinicopathological characteristics of the 72 enrolled patients. Among the 72 patients, mrEMVI was observed in 25 patients (34.7%).

**Table 2:** Clinicopathological characteristics and comparison of clinicopathological characteristics with mrEMVI status

Clinicopathological characteristics	Total	mrEMVI (−)	mrEMVI (+)	*P*
	*n* = 72 (100%)	*n* = 47 (65.3%)	*n* = 25 (34.7%)	
Age, mean ± SD	60 ± 10	61 ± 10	58 ± 10	0.214^a^
Gender				0.537^b^
Male	41 (56.9%)	28 (59.6%)	13 (52.0%)	
Female	31 (43.1%)	19 (40.4%)	12 (48.0%)	
Tumour location^#^				0.355^b^
Lower	13 (18.1%)	10 (21.3%)	3 (12.0%)	
Middle	32 (44.4%)	22 (46.8%)	10 (40.0%)	
Upper	27 (37.5%)	15 (31.9%)	12 (48.0%)	
Tumour size (cm), median (range)	3.0 (0.3–6.0)	2.9 (0.3–6.0)	3.5 (1.8–6.0)	0.011^c^*
Differentiation				0.234^d^
Well	1 (1.4%)	1 (2.1%)	0 (0.0%)	
Moderate	60 (83.3%)	41 (87.2%)	19 (76.0%)	
Poor	11 (15.3%)	5 (10.6%)	6 (24.0%)	
Pathological T stage				0.010^b^*
T1–2	19 (26.4%)	17 (36.2%)	2 (8.0%)	
T3–4	53 (73.6%)	30 (63.8%)	23 (92.0%)	
Pathological N stage				0.001^b^*
N0	36 (50.0%)	30 (63.8%)	6 (24.0%)	
N1–2	36 (50.0%)	17 (36.2%)	19 (76.0%)	
Synchronous distant metastasis				0.426^b^
Negative	51 (91.1%)	35 (94.6%)	16 (84.2%)	
Positive	5 (8.9%)	2 (5.4%)	3 (15.8%)	

*mrEMVI* magnetic resonance imaging-detected extramural vascular invasion, *SD* standard deviation, *T* tumour, *N* node

^#^According to the distance from the most caudal border of the rectal tumour to the anal verge on MRI: lower, < 5 cm; middle 5–10 cm; upper, > 10 cm

^a^independent samples *t*-test, ^b^*χ*^*2*^ test, ^c^Mann-Whitney *U* test, ^d^Fisher’s exact test, *indicates significant difference

The tumour size was significantly larger in mrEMVI-positive group (2.9 cm vs. 3.5 cm, *P* = 0.011). Higher pathological tumour stage (T3–4) was significantly more frequent in the mrEMVI-positive group (63.8% vs. 92.0%, *P* = 0.010). mrEMVI-positive status was associated with increased regional nodal metastases when compared with mrEMVI-negative status (36.2% vs. 76.0%, *P* = 0.001).

There was no significant difference in tumour location, differentiation or synchronous distant metastasis between different mrEMVI statuses (*P* = 0.355, 0.234 and 0.426, respectively). Among 56 patients with at least 6-month follow-up after the initial diagnosis, distant metastases to liver (3 patients) and lung (1 patient) were noted at the initial diagnostic workup, and one patient newly developed distant lymph node metastases within the 6-month follow-up.

### mrEMVI status compared with DCE-MRI parameters

**Fig. 4:**
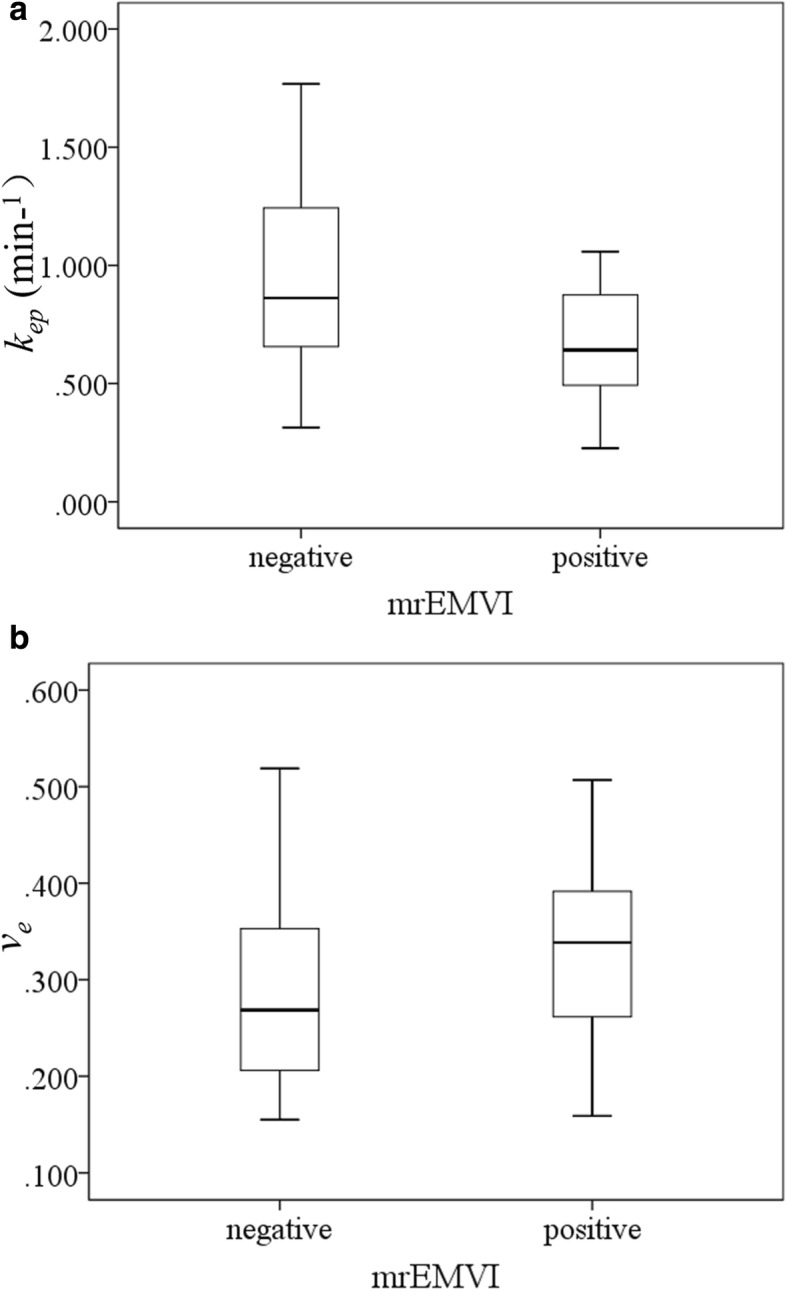
Boxplots showed the relationships between mrEMVI status and *k*_*ep*_ (**a**) and *v*_*e*_ (**b**) values. Lower *k*_*ep*_ values were noted among mrEMVI-positive patients than in mrEMVI-negative patients, while *v*_*e*_ values in the mrEMVI-positive group were significantly higher than those in the mrEMVI-negative group

There was no significant difference between mrEMVI-negative and -positive patients regarding *K*^*trans*^ values (*P* = 0.390). However, the *k*_*ep*_ values in the mrEMVI-positive group (0.710 ± 0.337 min^− 1^) were significantly lower than those in the mrEMVI-negative group (0.938 ± 0.364 min^− 1^) (*P* = 0.012). The *v*_*e*_ values in the mrEMVI-positive group (0.344 ± 0.101) were significantly higher than those in the mrEMVI-negative group (0.288 ± 0.094) (*P* = 0.021) (Table 3, Fig. 4).

**Table 3:** DCE-MRI parameters in patients with different mrEMVI statuses

	Total	mrEMVI (−)	mrEMVI (+)	*P*
	*n* = 72 (100%)	*n* = 47 (65.3%)	*n* = 25 (34.7%)	
*K*^*trans*^ (min^−1^)	0.231 ± 0.099	0.238 ± 0.104	0.217 ± 0.089	0.390^a^
*k*_*ep*_ (min^−1^)	0.859 ± 0.369	0.938 ± 0.364	0.710 ± 0.337	0.012^a^*
*v*_*e*_	0.307 ± 0.099	0.288 ± 0.094	0.344 ± 0.101	0.021^a^*

*DCE-MRI* dynamic contrast-enhanced magnetic resonance imaging, *mrEMVI* magnetic resonance imaging-detected extramural vascular invasion, *K*^*trans*^ volume transfer constant between blood plasma and extravascular extracellular space (EES), *k*_*ep*_ rate constant between EES and blood plasma, *v*_*e*_ volume of EES per unit volume of tissue

^a^independent samples *t*-test, *indicates a significant difference

### Interobserver agreement

There were nine discrepant cases for mrEMVI status evaluation between the two radiologists. The interobserver agreement for mrEMVI status evaluation between the two radiologists was good (*k* = 0.74, [95% CI: 0.58–0.90]).

**Fig. 5:**
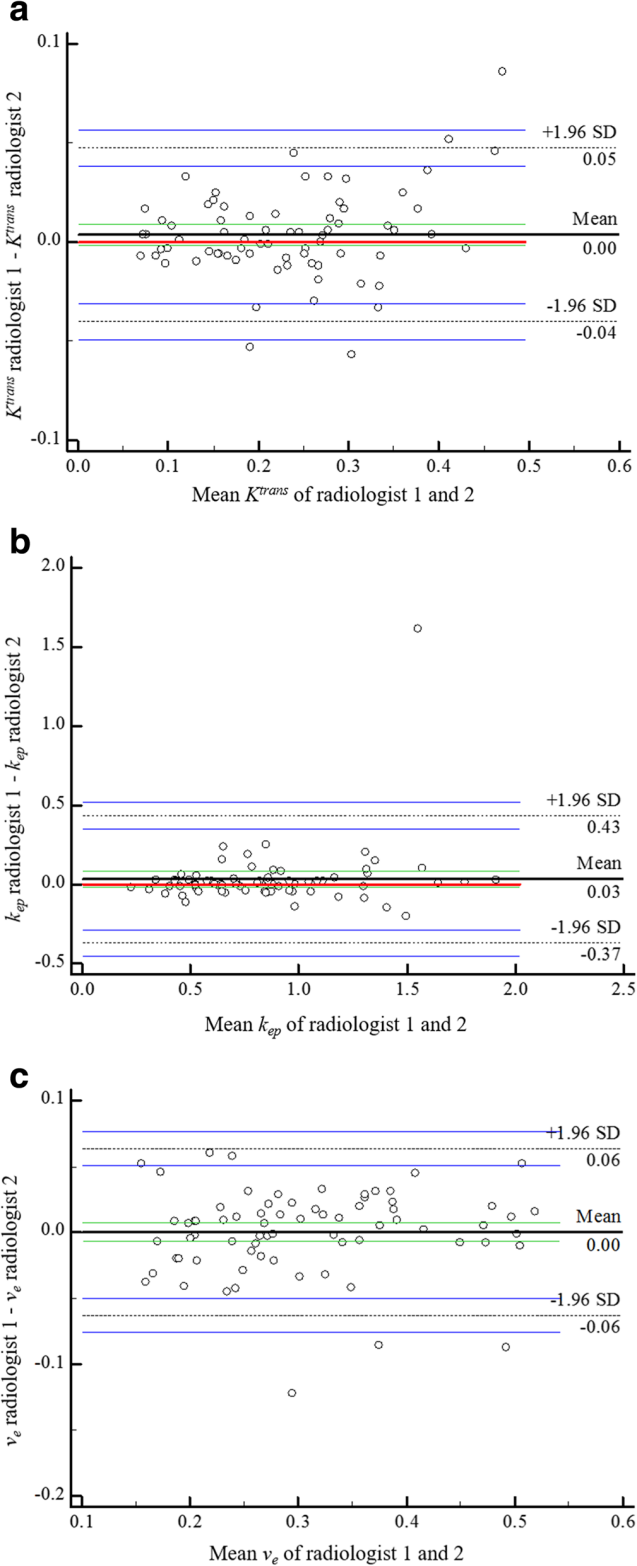
Bland-Altman plots for interobserver reproducibility for whole tumour volume DCE-MRI quantitative perfusion parameters measurements. Bland-Altman plots of the mean *K*^*trans*^ (**a**), *k*_*ep*_ (**b**) and *v*_*e*_ (**c**) values of the two radiologists (x-axis) against the difference between the two radiologists (y-axis). The red continuous line represents the equality (difference = 0); the black continuous line represents the average difference (bias) between the two radiologists; the green continuous lines represent the 95% CI of the average difference; the black dashed lines represent the limits of agreement; the blue continuous lines represent the 95% CI of limits of agreement

Interobserver reproducibility was excellent for *K*^*trans*^, *k*_*ep*_ and *v*_*e*_ values measurements (ICC = 0.97, [95% CI: 0.96–0.98], 0.86, [95% CI: 0.78–0.91] and 0.95, [95% CI: 0.92–0.97], respectively). Figure 5 displays the Bland-Altman plots for the DCE-MRI quantitative perfusion parameters measurements.

## Discussion

In the present study, we evaluated the association between mrEMVI status and tumour microcirculation in rectal adenocarcinoma. No significant difference was found in the DCE-MRI parameter *K*^*trans*^ between mrEMVI-negative and mrEMVI-positive patients, whereas the DCE-MRI parameters *k*_*ep*_ and *v*_*e*_ were revealed to be associated with mrEMVI status. It is interesting to note that mrEMVI-positive patients with rectal adenocarcinoma had significantly lower *k*_*ep*_ and higher *v*_*e*_ values than mrEMVI-negative patients.

It is now well established that angiogenesis is essential for tumour growth, invasion and lymphatic and haematogenous spread of tumour cells [11]. Newly formed capillaries are characterized by tortuous architecture and increased permeability compared with mature vessels; thus, they can be easily penetrated by tumour cells [16, 17]. mrEMVI, which is associated with a worse outcome, is an imaging indicator of tumour progression [10, 22]. In terms of biological explanations, we propose that tumour cells within intratumoural vessels, with further development, may extend into vessels outside the rectal wall to form EMVI [10, 23]. Thus, it can be presumed that the invasive degree of neovascularization is significantly different between mrEMVI-negative and mrEMVI-positive patients and that this alteration induces differences in the microcirculatory environment in tumour tissues. Quantitative perfusion parameters from DCE-MRI, a functional imaging method, provide information regarding related pathophysiological properties in vivo. Notably, mrEMVI-positive patients had significantly lower *k*_*ep*_ values. The *k*_*ep*_ value equals the flux rate of contrast agent from the EES to plasma, which depends largely on capillary permeability and the permeability surface area [15]. Based on the above hypothesis, the presence of a considerable number of tumour cells within the tumour vessels decreases the permeability surface area and thereby decreases the *k*_*ep*_ value in mrEMVI-positive patients. Clearly, a cluster of invasive tumour cells might cause decreased blood supply in the corresponding area due to a reduced permeability surface area, leading to an increased degree of tissue hypoxia and microscopic necrosis and, thus, an enlarged EES. The *v*_*e*_ value represents the fractional volume of the EES, which is affected by the integrated effects of cell proliferation and necrosis [24]. Thus, it was not unexpected that the *v*_*e*_ value was significantly higher in mrEMVI-positive patients. Yeo et al. [25] also demonstrated that microscopic internal necrotic areas showed a decreased *k*_*ep*_ value. In addition, it has been shown that the loss of function of cell-cell adhesion molecules, a pivotal step in tumour progression, leads to a larger interstitial space, which is reflected by a higher *v*_*e*_ value [26, 27].

The *K*^*trans*^ value represents the transfer rate from plasma to the EES, which has a mixed microcirculatory blood flow and permeability weighting [15, 28]. Although a mixed situation occurs most commonly, there is an overall trend of a dominating effect of flow in untreated tumours [29]. Theoretically, a cluster of tumour cells within vessels would decrease the local microcirculatory blood flow. In this study, as expected, there was a tendency towards a lower *K*^*trans*^ value in the mrEMVI-positive group, but the trend was nonsignificant. A possible explanation for this might be that the *K*^*trans*^ value is also impacted by blood perfusion, including cardiac output, hypertension and the circulatory system of an individual, which thus produces greater individual variation than other quantitative parameters. By comparison, *k*_*ep*_ is only influenced by the contrast concentration and fractional volumes in the tumour EES and might more accurately mirror the capillary permeability [30]. The results might also be limited by the complexity of the underlying pathophysiology.

A recent study also showed that primary rectal cancer blood flow measured by DCE-MRI was significantly lower in patients with metastatic nodes, which also represents an unfavourable prognosis [28]. However, regarding the investigation by Yu et al. [26], lymphovascular invasion was correlated with higher *K*^*trans*^, *k*_*ep*_ and *v*_*e*_ values. This inconsistency may be because lymphovascular invasion may be a different entity from EMVI. In addition, quantitative perfusion parameters from DCE-MRI primarily characterize angiogenesis rather than lymphangiogenesis. Furthermore, tumour development is a complex and heterogeneous process [31]. In addition, the inconsistency of DCE-MRI protocols could also affect the results to some extent.

mrEMVI is more likely to occur with more locally advanced tumours in rectal cancer [32]. Thus, it is not surprising that larger tumour size, higher pathological tumour stage and increased metastatic regional nodes were more common in mrEMVI-positive patients. Although nodal status is vital for prognosis and treatment decision-making in rectal cancer, all kinds of imaging methods have low discriminant accuracy for nodal metastases [33]. Therefore, from a practical perspective, the presence of mrEMVI can alert radiologists to carefully scrutinize the MRI for evidence of nodal involvement. However, our study has shown that mrEMVI status was not significantly associated with synchronous distant metastasis in rectal cancer. This outcome was contrary to that of Sohn et al. [22] who found that mrEMVI was an independent significant risk factor for synchronous distant metastasis in rectal cancer. A possible explanation for this might be differences in the patient populations of the two studies. Patients who underwent curative surgery with or without neoadjuvant treatment and only underwent chemotherapy or chemoradiotherapy were enrolled in their study; however, we only enrolled patients who proceeded directly to surgery. The patients who underwent surgery only have a low likelihood of synchronous distant metastasis, potentially influencing the relationship between mrEMVI status and synchronous distant metastasis.

Our study is hindered by a few limitations. First, mrEMVI was not directly compared with histopathology results. However, the reported incidence of EMVI on histopathology is variable, impairing the prognostic predictive value of EMVI. Although tumour deposits within small vessels are still beyond the spatial resolution of current MRI techniques [8], patients with histologically confirmed EMVI involving small vessels are the same as those with no demonstrable vascular invasion, with fewer clinical implications [22, 34]. Second, not every patient in this study underwent contrast-enhanced liver MRI or FDG-PET/CT, and it is possible that some synchronous distant metastases were missed. Lastly, the relatively small sample size did not allow for substratification of outcomes based on mrEMVI scores and different histopathological types of rectal cancer.

## Conclusions

This preliminary study suggests that the microcirculation of rectal adenocarcinoma in mrEMVI-positive patients is altered, with significantly lower *k*_*ep*_ and higher *v*_*e*_ values, enabling DCE-MRI parameters to serve as an additional tool for potentially predicting prognosis for mrEMVI-positive patients with rectal adenocarcinoma. In addition, larger tumour size, higher pathological tumour stage and metastatic regional nodes were more common in mrEMVI-positive patients.

## Data Availability

The datasets used and/or analysed during the current study are available from the corresponding author on reasonable request.

## Abbreviations

AIF: Arterial input function
CI: Confidence interval
CT: Computed tomography
DCE-MRI: Dynamic contrast-enhanced MRI
EES: Extravascular extracellular space
EMVI: Extramural vascular invasion
FDG-PET/CT: Fludeoxyglucose-positron emission tomography/computed tomography
FOV: Field of view
ICC: Intraclass correlation coefficient
*k*_*ep*_: Rate constant between EES and blood plasma
*K*^*trans*^: Volume transfer constant between blood plasma and EES
mrEMVI: MRI-detected extramural vascular invasion
MRI: Magnetic resonance imaging
MVD: Microvessel density
ROI: Region of interest
SD: Standard deviation
T2WI: T2-weighted imaging
TE: Echo time
TR: Repetition time
TWIST: Time-resolved technique with interleaved stochastic trajectories
*v*_*e*_: Volume of EES per unit volume of tissue
VEGF: Vascular endothelial growth factor
VIBE: Volume-interpolated body examination
VOI: Volume of interest
